# Supplementary material for: A general hypergraph learning algorithm for drug multi-task predictions in micro-to-macro biomedical networks
Source: PLoS Comput Biol. 2023 Nov 13;19(11):e1011597. doi: 10.1371/journal.pcbi.1011597 (PMC10681315; doi:10.1371/journal.pcbi.1011597)
Supplement: S1 Text — Fig A. Dimensionality reduction of the drug representations learned from the 4 hypergraph branches. The figure is drawn by t-SNE and the color corresponds to different types of drug’s ATC primary codes. Table A. Abbreviation List.Table B. The prediction results of other evaluation indicators for HGDrug and three GNN baseline models on four drug interaction datasets. The best performance is marked in bold and the second best is underlined to facilitate reading. Table C. Ablation experiments explore the contribution of the four motifs related to fragments.Table D. The top 20 novel drug-drug similarity pairs of the 5 drugs from the DDiI task’ drug feature.Table E. The top 20 novel DDIs description details.Table F. The top 20 novel DDIs from HGDrug 2f.Table G. The top 20 novel DDIs from HGDrug 2m.Table H. The top 10 novel targets of paclitaxel.Table I. The top 10 novel side effects of paclitaxel. (PDF) [file pcbi.1011597.s001.pdf]

A general hypergraph learning algorithm for drug multi-task predictions in micro-to-macro biomedical networks

Supporting information

List of abbreviations S1 Table displays the abbreviation list in this study for ease of reading.

Abbreviation	Full Meaning	Abbreviation	Full Meaning
DSMN	drugSubstructures relationship into molecular interaction networks	DL	Deep Learning
GNNs	graph neural networks	DDIs	drug-drug interactions
DTIs	drug-target interactions	DDiIs	drug-disease interactions
DSIs	drug-side-effect interactions	DFIs	drug-fragment interactions
FFIs	fragment-fragment interactions	AUROC	the area under the receiver operating characteristic curve
AUPR	the area under the precision-recall curve	DRSS	drug related and have the same substructure
DISS	drug independent and have the same substructure	DRSM	drug related and have the same molecular interactions
DISM	drug independent and have the same molecular interactions	MCC	Matthews correlation coefficient

Table A. Abbreviation List.

Details about General Models Baselines **SVM** [1] (support vector machines) is the traditional binary classification machine learning algorithm.

Katz [2] is the path-based classic algorithm calculating similarities between nodes in a network for associations prediction.

Deepwalk [3] use random walks to capture network structural information and learn the representations of nodes in the network.

GCN [4] (graph convolutional networks) is a scalable implementation of graph convolution that can be used for semi-supervised learning with graph-structured data.

GAT [5] uses masked self-attention layers to solve the shortcomings of previous graph convolution-based or approximate graph neural network models.

SkipGNN [6] aggregate informations from direct interactions and second order interactions for molecular interaction association prediction.

In addition, the parameter settings for the three GNN-based models (GCN, GAT, and SkipGNN) follow the optimal settings mentioned in the SkipGNN study: learning rate is $5e-4$ using the Adam optimizer, mini-batch size is 256, dropout rate is 0.1. Furthermore, the depth of the neural network layer is set to 2, with the hidden size in the first layer is set 64 and hidden size in the second layer is set 16.

More evaluation metrics In the section, we evaluated the performance of the HGDrug model across all four drug-related tasks using the MCC, precision, recall and F1-score evaluation metrics. Additionally, we incorporated other benchmark models such as GCN, GAT, and the recently developed SkipGNN in a comprehensive comparison experiment. The results are shown in the S2 Table below, which demonstrates that our model has achieved superior performance on the newly evaluation metrics.

Eevaluation metrics	Model	DDIs	DTIs	DDiIs	DSIs
**MCC**	GCN	<u>0.574</u>	<u>0.611</u>	<u>0.560</u>	0.734
	GAT	0.541	0.576	0.490	0.740
	SkipGNN	0.570	0.575	0.532	<u>0.749</u>
	**HGDrug**	**0.855**	**0.673**	**0.781**	**0.814**
**Precision**	GCN	0.750	<u>0.888</u>	**0.845**	<u>0.901</u>
	GAT	0.720	0.805	0.762	0.876
	SkipGNN	<u>0.760</u>	0.843	0.824	0.887
	**HGDrug**	**0.922**	**0.952**	<u>0.826</u>	**0.913**
**Recall**	GCN	0.855	0.681	0.674	0.811
	GAT	<u>0.870</u>	**0.762**	<u>0.719</u>	<u>0.862</u>
	SkipGNN	0.830	0.697	0.677	0.857
	**HGDrug**	**0.934**	<u>0.678</u>	**0.822**	**0.899**
**F1**	GCN	<u>0.799</u>	0.771	0.749	0.857
	GAT	0.788	<u>0.783</u>	0.740	0.869
	SkipGNN	0.793	0.763	<u>0.744</u>	<u>0.872</u>
	**HGDrug**	**0.928**	**0.792**	**0.879**	**0.906**

Table B. The prediction results of other evaluation indicators for HGDrug and three GNN baseline models on four drug interaction datasets. The best performance is marked in bold and the second best is underlined to facilitate reading.

More ablation studies To more thoroughly evaluate the significance of utilizing fragments as a basis for hypergraph construction in drug feature learning, we conducted additional ablation experiments on the DDI task. This allowed us to analyze the contributions of the four specific motifs (M_2, M_3, M_5, M_6) related to fragments in influencing the prediction outcomes. The experimental results are reported in S3 Table. It can be observed that the removal of the M_2 motif has the least impact on the outcomes. This can be attributed to the fact that drugs sharing the same fragment in the M2 motif are directly related, meaning that the information encapsulated within this fragment is already available in existing biomolecular databases. Consequently, omitting this motif during hypergraph construction has minimal impact on the results. Conversely, other motifs facilitate the model in discerning higher-order associations between drugs; therefore, their removal will have a greater impact on the predictive performance of the model.

	**Remove** M_2	**Remove** M_3	**Remove** M_5	**Remove** M_6	No remove
**AUROC**	0.973	0.970	0.970	0.970	0.976
**AUPR**	0.975	0.971	0.970	0.971	0.977

Table C. Ablation experiments explore the contribution of the four motifs related to fragments.

Visualization details We examine the drug features learned from 4 hypergraph branches by using t-SNE (t-distributed stochastic neighbor embedding), a non-linear dimensionality reduction method that embeds similar points in the high-dimensional space as points close in two dimensions. Each point in the figure represents a drug node projected from the 100-dimensional feature vectors extracted from the hypergraph branches process. We download the Anatomical Therapeutic Chemical (ATC) classification data of drug from the DrugBank (https://go.drugbank.com/). We match them with our drug data and classify them by ATC primary codes. Finally, 1361 classifications of diseases were obtained. We selected those with more than 100 drug in

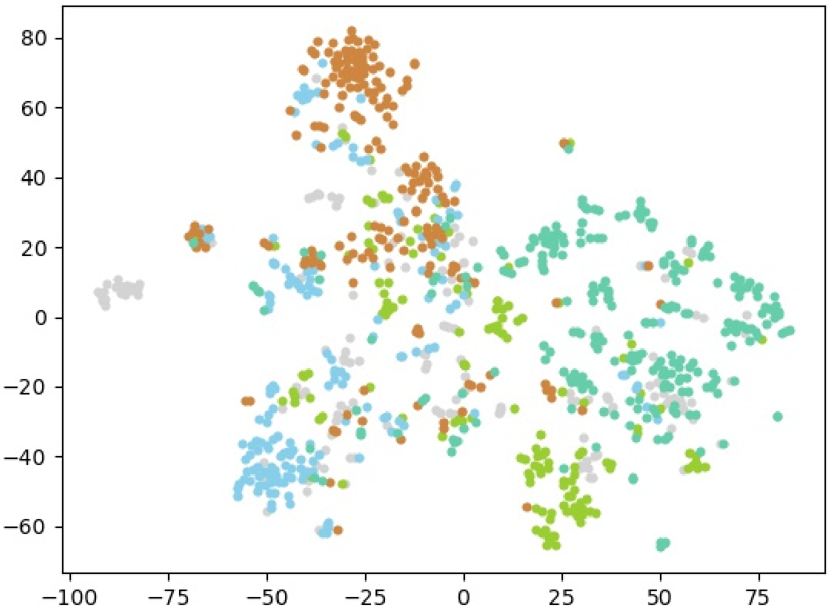

Fig A. Dimensionality reduction of the drug representations learned from the 4 hypergraph branches. The figure is drawn by t-SNE and the color corresponds to different types of drug's ATC primary codes.

the category for dimensionality reduction visualization (S1 Fig). The same type of nodes is highlighted with the same color. In the figure, a clustering with clear boundaries between different color groups proves the quality of the drug features representations from hypergraph branches.

Rank	Drug 1	Drug 2	Rank	Drug 1	Drug 2
1	DB01331 Cefoxitin	DB01330 Cefotetan	11	DB01331 Cefoxitin	DB00493 Cefotaxime
2	DB01413 Cefepime	DB01330 Cefotetan	12	DB00535 Cefdinir	DB00833 Cefaclor
3	DB01331 Cefoxitin	DB00766 Clavulanate	13	DB00833 Cefaclor	DB00535 Cefdinir
4	DB01413 Cefepime	DB00766 Clavulanate	14	DB00535 Cefdinir	DB01330 Cefotetan
5	DB01331 Cefoxitin	DB01413 Cefepime	15	DB00833 Cefaclor	DB01413 Cefepime
6	DB01413 Cefepime	DB01331 Cefoxitin	16	DB01413 Cefepime	DB00833 Cefaclor
7	DB01413 Cefepime	DB00485 Dicloxacillin	17	DB00535 Cefdinir	DB01413 Cefepime
8	DB01331 Cefoxitin	DB00485 Dicloxacillin	18	DB01413 Cefepime	DB00535 Cefdinir
9	DB01413 Cefepime	DB00948 Mezlocillin	19	DB01331 Cefoxitin	DB00438 Ceftazidime
10	DB00833 Cefaclor	DB01416 Cefpodoxime	20	DB00535 Cefdinir	DB00766 Clavulanate

Table D. The top 20 novel drug-drug similarity pairs of the 5 drugs from the DDiI task' drug feature.

Novel potentially interactions Analysis Supplementary Data

Rank	Drug 1	Drug 2	DrugBank interactions
1	Magnesium salicylate	Liothyronine	Magnesium salicylate may decrease the excretion rate of Liothyronine which could result in a higher serum level.
2	Aliskiren	Dipyridamole	Dipyridamole may increase the antihypertensive activities of Aliskiren.
3	Silodosin	Diltiazem	The metabolism of Silodosin can be decreased when combined with Diltiazem.
4	Calcium carbonate	Levothyroxine	Calcium carbonate can cause a decrease in the absorption of Levothyroxine resulting in a reduced serum concentration and potentially a decrease in efficacy.
5	Netupitant	Zopiclone	The metabolism of Zopiclone can be decreased when combined with Netupitant.
6	Calcium carbonate	Alfacalcidol	The risk or severity of adverse effects can be increased when Alfacalcidol is combined with Calcium carbonate.
7	Acenocoumarol	Ciprofloxacin	The serum concentration of Acenocoumarol can be increased when it is combined with Ciprofloxacin.
8	Dabrafenib	Propafenone	The serum concentration of Propafenone can be decreased when it is combined with Dabrafenib.
9	Dabrafenib	Domperidone	The serum concentration of Domperidone can be decreased when it is combined with Dabrafenib.
10	Dasatinib	Escitalopram	The risk or severity of QTc prolongation can be increased when Dasatinib is combined with Escitalopram.
11	Picosulfuric acid	Mycophenolate mofetil	The therapeutic efficacy of Picosulfuric acid can be decreased when used in combination with Mycophenolate mofetil.
12	Ceritinib	Fosaprepitant	The metabolism of Ceritinib can be increased when combined with Fosaprepitant.
13	Dabrafenib	Budesonide	The serum concentration of Budesonide can be decreased when it is combined with Dabrafenib.
14	Canagliflozin	Sitagliptin	Canagliflozin may increase the hypoglycemic activities of Sitagliptin.
15	Metamizole	Adalimumab	None.
16	Simeprevir	Propafenone	The metabolism of Propafenone can be decreased when combined with Simeprevir.
17	Lithium cation	Amiloride	None.
18	Dasatinib	Bortezomib	The metabolism of Bortezomib can be decreased when combined with Dasatinib.
19	Dabrafenib	Bromocriptine	The serum concentration of Bromocriptine can be decreased when it is combined with Dabrafenib.
20	Fosphenytoin	Chloramphenicol	The serum concentration of Fosphenytoin can be increased when it is combined with Chloramphenicol.

Table E. The top 20 novel DDIs description details.

Rank	Drug 1	Drug 2	DrugBank interactions
1	Silodosin	Diltiazem	The metabolism of Silodosin can be decreased when combined with Diltiazem.
2	Dabrafenib	Colchicine	The metabolism of Colchicine can be decreased when combined with Dabrafenib.
3	Salicylic acid	Desogestrel	The metabolism of Salicylic acid can be decreased when combined with Desogestrel.
4	Palbociclib	Imatinib	The serum concentration of Palbociclib can be increased when it is combined with Imatinib.
5	Ceritinib	Atorvastatin	The metabolism of Atorvastatin can be decreased when combined with Ceritinib.
6	Riociguat	Amiodarone	The serum concentration of Riociguat can be increased when it is combined with Amiodarone.
7	Canagliflozin	Sitagliptin	Canagliflozin may increase the hypoglycemic activities of Sitagliptin.
8	Dabrafenib	Rifampicin	The serum concentration of Dabrafenib can be decreased when it is combined with Rifampicin.
9	Tesmilifene	Sunitinib	The metabolism of Sunitinib can be increased when combined with Tesmilifene.
10	Netupitant	Zopiclone	The metabolism of Zopiclone can be decreased when combined with Netupitant.
11	Salicylic acid	Megestrol acetate	Salicylic acid may decrease the excretion rate of Megestrol acetate which could result in a higher serum level.
12	Cobicistat	Trazodone	The serum concentration of Trazodone can be increased when it is combined with Cobicistat.
13	Netupitant	Everolimus	The metabolism of Everolimus can be decreased when combined with Netupitant.
14	Ceritinib	Topotecan	The serum concentration of Topotecan can be increased when it is combined with Ceritinib.
15	Magnesium salicylate	Liothyronine	Magnesium salicylate may decrease the excretion rate of Liothyronine which could result in a higher serum level.
16	Salicylic acid	Ethynodiol diacetate	None.
17	Dabrafenib	Propafenone	The serum concentration of Propafenone can be decreased when it is combined with Dabrafenib.
18	Mirabegron	Pimozide	The serum concentration of Pimozide can be increased when it is combined with Mirabegron.
19	Aliskiren	Dipyridamole	Dipyridamole may increase the antihypertensive activities of Aliskiren.
20	Dabrafenib	Domperidone	The serum concentration of Domperidone can be decreased when it is combined with Dabrafenib.

Table F. The top 20 novel DDIs from HGDrug_2f.

Rank	Drug 1	Drug 2	DrugBank interactions
1	Palbociclib	Imatinib	The serum concentration of Palbociclib can be increased when it is combined with Imatinib.
2	Metamizole	Etanercept	None.
3	Dasatinib	Escitalopram	The risk or severity of QTc prolongation can be increased when Dasatinib is combined with Escitalopram.
4	Metamizole	Adalimumab	None.
5	Dasatinib	Imatinib	The serum concentration of Dasatinib can be increased when it is combined with Imatinib.
6	Fosaprepitant	Docetaxel	The metabolism of Docetaxel can be increased when combined with Fosaprepitant.
7	Doxofylline	Calcium acetate	None.
8	Salsalate	Icosapent	The risk or severity of adverse effects can be increased when Icosapent is combined with Salsalate.
9	Cathinone	Maprotiline	Cathinone may increase the central nervous system depressant (CNS depressant) activities of Maprotiline.
10	Mirabegron	Pimozide	The serum concentration of Pimozide can be increased when it is combined with Mirabegron.
11	Obinutuzumab	Dexmedetomidine	The risk or severity of adverse effects can be increased when Dexmedetomidine is combined with Obinutuzumab.
12	Metamizole	Antithymocyte immunoglobulin (rabbit)	None.
13	Canagliflozin	Sitagliptin	Canagliflozin may increase the hypoglycemic activities of Sitagliptin.
14	Vorapaxar	Deferasirox	The risk or severity of gastrointestinal bleeding can be increased when Vorapaxar is combined with Deferasirox.
15	Moxonidine	Dexmedetomidine	Dexmedetomidine may decrease the antihypertensive activities of Moxonidine.
16	Moxonidine	Carteolol	Carteolol may increase the hypotensive activities of Moxonidine.
17	Apixaban	Celecoxib	The risk or severity of bleeding and hemorrhage can be increased when Celecoxib is combined with Apixaban.
18	Metamizole	Basiliximab	None.
19	Ceritinib	Bromocriptine	The therapeutic efficacy of Bromocriptine can be decreased when used in combination with Ceritinib.
20	Canagliflozin	Rosiglitazone	Canagliflozin may increase the hypoglycemic activities of Rosiglitazone.

Table G. The top 20 novel DDIs from HGDrug_2m.

Rank	UniProtKB ID ID	Gene Name	Description
1	P03372	ESR1	[7]
2	P11388	TOP2A	[8]
3	P35354	PTGS2	[9]
4	P10275	AR	[10]
5	P68366	TUBA4A	[11]
6	P78559	MAP1A	None
7	P07437	TUBB	[12]
8	P04150	NR3C1	None
9	P22309	UGT1A1	None
10	P06401	PGR	[13]

Table H. The top 10 novel targets of paclitaxel.

Rank	Side effect ID	Side effect Name	Description
1	C0392699	Dysaesthesia	[14]
2	C0031256	Petechiae	[15]
3	C0428974	Arrhythmia supraventricular	[16]
4	C0006266	Bronchospasm	[17]
5	C0019521	Hiccups	[18]
6	C0542571	Face oedema	[19]
7	C0349506	Photosensitivity	[20]
8	C0004239	Atrial flutter	[21]
9	C0021368	Inflammation	[22]
10	C1510410	Parosmia	None

Table I. The top 10 novel side effects of paclitaxel.

References

1. Cortes C, Vapnik V. Support-vector networks. Machine learning. 1995;20(3):273–297.

2. Chen X, Huang YA, You ZH, Yan GY, Wang XS. A novel approach based on KATZ measure to predict associations of human microbiota with non-infectious diseases. Bioinformatics. 2017;33(5):733–739.

3. Perozzi B, Al-Rfou R, Skiena S. Deepwalk: Online learning of social representations. In: Proceedings of the 20th ACM SIGKDD international conference on Knowledge discovery and data mining; 2014. p. 701–710.

4. Henaff M, Bruna J, LeCun Y. Deep convolutional networks on graph-structured data. arXiv preprint arXiv:150605163. 2015;.

5. Veličković P, Cucurull G, Casanova A, Romero A, Lio P, Bengio Y. Graph attention networks. arXiv preprint arXiv:171010903. 2017;.

6. Huang K, Xiao C, Glass LM, Zitnik M, Sun J. SkipGNN: predicting molecular interactions with skip-graph networks. Scientific reports. 2020;10(1):1–16.

7. Flanagan J, Wilhelm-Benartzi C, Metcalf M, Kaye S, Brown R. Association of somatic DNA methylation variability with progression-free survival and toxicity in ovarian cancer patients. Annals of oncology. 2013;24(11):2813–2818.

8. Zhang S, Jiang H, Xu Z, Jiang Y, She Y, Huang X, et al. The resistance of esophageal cancer cells to paclitaxel can be reduced by the knockdown of long

noncoding RNA DDX11-AS1 through TAF1/TOP2A inhibition. American journal of cancer research. 2019;9(10):2233.

9. Im JH, Kim SJ. Paclitaxel stimulates cyclooxygenase-2 expression via MAP kinase pathway in rabbit articular chondrocytes. Biomedical Science Letters. 2009;15(2):141–146.

10. Gan L, Chen S, Wang Y, Watahiki A, Bohrer L, Sun Z, et al. Inhibition of the androgen receptor as a novel mechanism of taxol chemotherapy in prostate cancer. Cancer research. 2009;69(21):8386–8394.

11. Zheng W, Xu S. Analysis of differential expression proteins of paclitaxel-treated lung adenocarcinoma cell A549 using tandem mass tag-based quantitative proteomics. OncoTargets and therapy. 2020;13:10297.

12. Narvi E, Jaakkola K, Winsel S, Oetken-Lindholm C, Halonen P, Kallio L, et al. Altered TUBB3 expression contributes to the epothilone response of mitotic cells. British journal of cancer. 2013;108(1):82–90.

13. Schmidt M, Bremer E, Hasenclever D, Victor A, Gehrmann M, Steiner E, et al. Role of the progesterone receptor for paclitaxel resistance in primary breast cancer. British journal of cancer. 2007;96(2):241–247.

14. Andoh T, Kobayashi N, Kuraishi Y. Prophylactic repetitive shakuyakukanzoto treatment inhibits paclitaxel-induced mechanical allodynia in mice via peripheral effects. Traditional & Kampo Medicine. 2016;3(1):71–74.

15. Üyetürk Ü, Arslan SH, Yüksel MK, Altuntas F. Paclitaxel therapy and immune thrombocytopenic purpura: coincidence or association?/Paclitaxel tedavisi ve immun trombositopeni: rastlanti mi, iliski mi? Turkish Journal of Haematology. 2011;28(2):151.

16. Van Herpen C, Van Hoesel Q, Punt C. Paclitaxel-induced severe hypersensitivity reaction occurring as a late toxicity. 1995;.

17. Otani IM, Lax T, Long AA, Slawski BR, Camargo Jr CA, Banerji A. Utility of risk stratification for paclitaxel hypersensitivity reactions. The Journal of Allergy and Clinical Immunology: In Practice. 2018;6(4):1266–1273.

18. Zhou JN, Huang XE, Ye Z, Li C, Zhang Q, Lin Y, et al. Weekly paclitaxel/Docetaxel combined with a paltinum in the treatment of advanced non-samll cell lung cancer: a study on efficacy, safety and pre-medication. Asian Pac J Cancer Prev. 2009;10(6):1147–1150.

19. Kim R, Osaki A, Toge T. Feasibility and therapeutic efficacy of weekly 1-h low-dose paclitaxel infusion for relapsed breast cancer. Oncology reports. 2003;10(1):145–150.

20. Beutler BD, Cohen PR. Nab-paclitaxel-associated photosensitivity: report in a woman with non-small cell lung cancer and review of taxane-related photodermatoses. Dermatology Practical & Conceptual. 2015; p. 121–124.

21. Kamineni P, Prakasa K, Hasan SP, Akula R, Dawkins F. Cardiotoxicities of paclitaxel in African Americans. Journal of the National Medical Association. 2003;95(10):977.

22. Xu Y, Jiang Z, Chen X. Mechanisms underlying paclitaxel-induced neuropathic pain: Channels, inflammation and immune regulations. European Journal of Pharmacology. 2022; p. 175288.
