# Supplementary material for: A general hypergraph learning algorithm for drug multi-task predictions in micro-to-macro biomedical networks
Source: PLoS Comput Biol. 2023 Nov 13;19(11):e1011597. doi: 10.1371/journal.pcbi.1011597 (PMC10681315; doi:10.1371/journal.pcbi.1011597)
Supplement: Attachment — Submitted filename: Response to Reviewers.docx [file pcbi.1011597.s002.docx]

***Reviewer #1***

*Jin et al. reported in this manuscript a hypergraph-based deep learning framework tailed for drug-related predictions using fragments of drug molecules. In particular, BRICS decomposition is used to partition each small-molecule drug into functional group fragments at a variety of levels, and the interference is performed based on fragments instead of whole molecules. Most importantly, the deployment of hypergraphs by the authors is suitable and imperative for accommodating the higher-order relationships between fragments/motifs. The authors demonstrated that such an approach can be successfully used to perform interference for drug-drug interactions, drug-target interactions, drug-disease relationships, and drug side-effect interactions.* *The manuscript is in general well-written, and should be suitable for PLOS Comp. Biol. It can be improved by addressing the following comments.*

**Response:** We thank the reviewer for the great summary and his/her positive support on our manuscript.

*1)* *The section discussing implementation specifics should elucidate the number of learnable parameters in the neural network (NN) models. This should be in tandem with the information about training parameters and the utilized platform.*

**Response:** Thanks for your valuable comments. We made some revisions :“ *The neural network model was developed and trained utilizing the TensorFlow framework, specifically version 2.2.0. The trainable parameters of our model consist of three parts: drug and drug-related node embeddings, gate parameters, and attention parameters. Regarding drug and drug-related node embeddings, the HGDrug only need to learn the 0-th layer drug embeddings* $\mathcal{D}_{0}\in\mathbb{R}^{b\times c}$ *and drug-related node embeddings* $\mathcal{M}_{0}\in\mathbb{R}^{t\times c}$*. In the HGDrug architecture, a total of nine gates are employed. Each of the gate has parameters of* $\left( c+1 \right)\times c$*, while the attention parameters are of the same size. For the definition of formula symbols, see Methods*”, and “we use the adaptive learning rate to train HGDrug with a initial learning rate 0.001, and all experiments are run up to 200 epochs”. We have added this description to the manuscript.

*2) To ensure a fair comparison, the inclusion of other* *evaluation metrics—such as MCC, Precision, Recall, and F1-score—is advised for all methods in comparison across all four drug-related tasks.*

**Response:** Thank you very much for your suggestion. For task-specific models, we observed that most studies primarily focus on the AUROC and AUPR metrics. Therefore, we decided not to adjust these task-specific models to incorporate new indicators for comparison. However, to address the reviewer's feedback, we evaluated the performance of the HGDrug model across all four drug-related tasks using the four suggested metrics. Additionally, we incorporated GNN benchmark models such as GCN, GAT, and the recently developed SkipGNN in a comprehensive comparison experiment. The results are shown in the table below, whichs demonstrates that our model has achieved superior performance on the newly evaluation metrics. We have added these results in the “Supporting information” for further reference.

| ***Eevaluation metrics*** | Model | **DDIs** | **DTIs** | **DDiIs** | **DSIs** |
| --- | --- | --- | --- | --- | --- |
| **MCC** | GCN | 0.574 | 0.611 | 0.560 | 0.734 |
|  | GAT | 0.541 | 0.576 | 0.490 | 0.740 |
|  | SkipGNN | 0.570 | 0.575 | 0.532 | 0.749 |
|  | **HGDrug** | **0.855** | **0.673** | **0.781** | **0.814** |
| **Precision** | GCN | 0.750 | 0.888 | **0.845** | 0.901 |
|  | GAT | 0.720 | 0.805 | 0.762 | 0.876 |
|  | SkipGNN | 0.760 | 0.843 | 0.824 | 0.887 |
|  | **HGDrug** | **0.922** | **0.952** | 0.826 | **0.913** |
| **Recall** | GCN | 0.855 | 0.681 | 0.674 | 0.811 |
|  | GAT | 0.870 | **0.762** | 0.719 | 0.862 |
|  | SkipGNN | 0.830 | 0.697 | 0.677 | 0.857 |
|  | **HGDrug** | **0.934** | 0.678 | **0.822** | **0.899** |
| **F1** | GCN | 0.799 | 0.771 | 0.749 | 0.857 |
|  | GAT | 0.788 | 0.783 | 0.740 | 0.869 |
|  | SkipGNN | 0.793 | 0.763 | 0.744 | 0.872 |
|  | **HGDrug** | **0.928** | **0.792** | **0.879** | **0.906** |

*3) The manuscript would benefit from a more comprehensive description of the baseline models, especially concerning* *the three GNN-based models. Given that the efficacy of NNs is closely tied to their configurations, the authors, at the very least, should specify the number of learnable weights in the baseline models in relation to their HGDrug models.*

**Response:** Thanks for your valuable comment. We have added the description of the baseline models in the “Details about General Models Baselines” section of Supporting information, as follows:“The parameter settings for the three GNN-based models (GCN, GAT, and SkipGNN) follow the optimal settings mentioned in the SkipGNN study: learning rate is $5e-4$ using the Adam optimizer, mini-batch size is 256, dropout rate is 0.1. Furthermore, the depth of the neural network layer is set to 2, with the hidden size in the first layer is set 64 and hidden size in the second layer is set 16.”

*4) I checked the corresponding* *github repository and found the introduction unclear. It would be beneficial to incorporate a comprehensive README section, detailing download instructions, software requirements, training protocols, and illustrative examples of outputs.*

**Response:** Thanks for your valuable comment, we've updated the *README* in our *GitHub* repository, which now includes information on environment downloads, download channels, training protocols, output content, and the required environment listed in the 'requirements.txt' file.

*5) Table 1 requires a more detailed explanation and* *description.*

**Response:** Thank you very much for your suggestion. We have added the detail description as follows:“ Statistical information for the four interactions datasets: the number of drugs, drug-related nodes, fragments and interaction pairs.”

*Besides, I'd like to draw attention to a potential typo error concerning the eighth affiliation of the corresponding author (X.L.). Please check whether it is "Zhejiang Lab" as opposed to "Zhijiang Lab".*

**Response:** Thanks for your careful review, we corrected the error.

***Reviewer #2:***

*Summary: The authors present a hypergraph learning algorithm named HGDrug for drug multi-task predictions. The algorithm incorporates drug-substructure relationships into molecular interaction networks to construct a drug-centric heterogeneous network. HGDrug captures high-order drug relations and fetches effective drug features using motif-driven hypergraphs and a self-supervised auxiliary task. This study demonstrates HGDrug achieves state-of-the-art performance and the ability to capture relations between drugs with the same functional groups. The proposed drug-substructure interaction networks can also improve the performance of existing network models for drug-related prediction tasks. The paper is well-written, and the method is clearly described. I only have a few comments and suggestions for the authors.*

**Response:** We thank the reviewer for the great summary and his/her positive support on our manuscript.

***Major:*** *The introduction does provide a clear objective of the study, which is to introduce a new hypergraph learning algorithm for drug multi-task predictions. However, the significance of this objective in the broader context of drug discovery could be elaborated upon more.
I suggest they give a deeper explanation about why their objective is challenged or crucial.
While the work does touch upon the importance of drug multi-task predictions, a more detailed motivation explaining the challenges in the current landscape would provide a stronger foundation for the study. The authors briefly mention terms like* *"**motif-driven hypergraphs" and "self-supervised auxiliary task". It would be better to give a brief explanation or reference for these terms in the introduction.*

**Response:** Thanks for your valuable comment. We have revised the section “Introduction” and added the references of "motif-driven hypergraphs" and "self-supervised auxiliary task". More explanations of " motif-driven hypergraphs " can be found in “Preliminary and problem formulation”.

*While the paper claims to introduce a novel approach by incorporating drug substructure information, it would be beneficial to provide a clearer distinction description between the proposed method and existing graph-based methods. For example, give more information to introduce the difference between DSMN and the heterogeneous-network-based methods.*
**Response:** Thank you very much for your suggestion. By treating the drug substructure, which contains pharmacophore information, as a node type and incorporating it into the heterogeneous network, we can construct a micro-to-macro drug-centric heterogeneous network (DSMN). Methods based on this type of network can more effectively capture the relationship between drugs that may have similar efficacy, thereby improving the prediction performance of drug-related tasks. We have incorporated relevant discussions in the section titled "Performance Comparison", and the experimental results also demonstrate that the consideration of drug substructure networks can enhance the performance of existing GNN models.

*I suggest the authors underscore the significance and benefits of their approach compared to traditional methods. Many researchers tend to favor established techniques unless the new method addresses specific challenges that traditional methods cannot overcome. Why is there a pressing need for another drug prediction algorithm?
What specific challenges does HGDrug address that others don't?*
**Response:** Thanks for your insightful comment. We have added related discussion in the section “Conclusion”, and revised this section title to “Discussion and conclusion”. Revised as follows:“ *The biomedical networks which contain domain knowledge and can be used for the prediction of various tasks such as drug repurposing and drug-drug interaction, however, chemical that play important role in drug properties is neglected in current biomedical networks. Furthermore, previous drug-related interactions predictions methods mostly rely on pairwise links heavily, without paying attention to local strong connections in complex biological networks, and cannot correlation between drugs and chemical structures.*”, “*Moreover, HGDrug is adept at handling all drug-related interaction task predictions, significantly minimizing the consumption of computing resources and facilitating a more convenient and efficient execution of related tasks.*”

*The work could benefit from better structuring, possibly with more defined subheadings, to guide the reader through different sections. I suggest the authors could summarize the section's conclusion as the subheading for readers’ better understanding.*

**Response:** Thanks for your valuable comment. We have revised some section's titles, for example: “*Performance of HGDrug on the cross-validation*”, “*Performance of HGDrug by ablation analysis*”, “*Discussion and conclusion*”.

*It would be beneficial to provide a* *detailed explanation for the operator “*$\delta\left\| \theta\right\|_{2}^{2}$*” in Formula 8.*
**Response:** Thanks for your valuable comment. We have added the detailed explanation for the operator “$\delta\left\| \theta\right\|_{2}^{2}$”, as follows:“ $\left\| \theta\right\|_{2}$ *is the* $L_{2}$ *norm of the parameter vector*”.

*For discussion
It would be better to highlight to emphasize the importance of the potential applications or implications of the HGDrug algorithm in real-world scenarios could be.
I suggest the authors have a* *discussion on the computational complexity of HGDrug, its scalability, and performance concerning dataset size would provide insights into its feasibility for large-scale applications.*

**Response:** Thank you very much for your suggestion and point out this. We have added the related discussion as follows:“ *Furthermore, the hypergraph-based method is not constrained by the size of the network data. It leverages existing network data to extract strong correlations between drugs, thereby enhancing the predictive accuracy in drug-related tasks. This approach allows for the expansion of calculations to larger network datasets without escalating computational complexity. Moreover, HGDrug is adept at handling all drug-related interaction task predictions, significantly minimizing the consumption of computing resources and facilitating a more convenient and efficient execution of related tasks.*”

***Minor:*** *Refining and condensing your writing will improve the paper's readability. For example: In the Definition 2 section, "The motif is a small pattern of interconnections occurring in complex networks at numbers that are significantly higher than those in randomized networks," consider revising to "**A motif is a recurring pattern of interconnections in complex networks, occurring more frequently than in randomized networks."*
**Response:** Thanks for your valuable comment. We have revised the description, as follows :“*A motif represents a small pattern of interconnections that occurs in complex networks at a frequency significantly higher than in randomized networks, thereby characterizing higher-order network structures*”.

*For references, ensure all references are up-to-date and relevant. Cross-check that all cited works are appropriately referenced within the text.
In this* *article, there are many abbreviations, it would be better to consider* *providing a list or* *table of* *abbreviations for quick reference.
The manuscript delves into technical details regarding* *hypergraphs, motifs, and heterogeneous networks. It would be helpful to provide more intuitive explanations or examples for readers less familiar with these concepts.*

**Response:** Thank you very much for your suggestion. We have meticulously reviewed all the works cited in the article to ensure appropriate citations and have incorporated additional relevant citations in response to feedback from other reviewers. The concepts of hypergraphs, motifs, and heterogeneous networks are elaborated upon in the 'Preliminary and Problem Formulation' section. In addition, we have added the abbreviation table in this study for ease of reading, see the Table S1 in “Supporting information”.

***Reviewer #3:***

*The manuscript describes a hypergraph learning model, namely HGDrug, which introduces drug substructure (functional group fragments) into biomedical networks for the first time. Specifically, the work constructs a drug-centric micro-to-macro heterogeneous network (termed DSMN) and presents a motif-driven hypergraph learning framework (termed HGDrug) with the self-supervised auxiliary task for drug multi-task interaction predictions. The method is evaluated by 4 benchmark tasks and demonstrates that it achieves highly accurate and robust predictions, outperforming 8 state-of-the-art task-specific models and 6 general-purpose conventional models. A specific case study also shows that HGDrug can learn the substructure information to improve the performance of drug repurposing. However, I have the following concerns.*

Response: We thank the reviewer for the great summary and his/her positive support on our manuscript.

Major comments:
*1、 Fragments increase the number of nodes and edges of these networks. For example, for the Drug-drug, Drug-target, Drug-disease, and Drug-side-effect datasets, the number of* *fragments is 4.42 times the number of nodes. Please discuss the balance between time and performance before and after adding fragments.*

Response: Thanks for your valuable comment. In this work, the fragments of the drug are only used to construct a hypergraph related to substructure information, which means that an increase in the number of fragments will not lead to an increase in the cost and time of model training. During the training process of the deep learning model, the input related to fragments is limited to the high-order relationships between drug-drug interactions obtained through fragments.

*2、 In the part of the “Ablation* *studies”, the author explored the influence of 4 hypergraph-based branches on the prediction performance of the model, but I noticed that each hypergraph branch also contains multiple network motifs to derive hypergraphs. I think the author should* *study the* *contribution of* *the four kinds of motifs (*$M_{2}$*,* $M_{3}$*,* $M_{5}$*,* $M_{6}$*) related to fragments to the prediction results, and further verify the importance of constructing hypergraphs based on fragments for drug feature learning.*

Response: Thanks for your valuable comment. To verify the contribution of the four motifs ($M_{2}$, $M_{3}$, $M_{5}$, $M_{6}$), we conducted ablation experiments within the DDI task, wherein each of these motifs was removed in turn. We have added these results in the “Supporting information”.

|  | Remove $M_{2}$ | Remove $M_{3}$ | Remove $M_{5}$ | Remove $M_{6}$ | No remove |
| --- | --- | --- | --- | --- | --- |
| AUROC | 0.973 | 0.970 | 0.970 | 0.970 | 0.976 |
| AUPR | 0.975 | 0.971 | 0.970 | 0.971 | 0.977 |

*3、 The work uses BRICS to* *decompose the SMILES sequences of drugs when acquiring DFIs and FFIs networks. As far as I know, there are many ways for SMILES decomposition, such as rdkit Recap, why did the author choose BRICS? BRICSDecompose should return a non-repeated fragments list after decomposition. How did the author achieve FFI?*

Response: Because the BRICS decomposition method retains the pharmacophore structure of the drug, which is central to the drug's efficacy. During the segmentation process, we didn't directly invoke the BRICSDecompose function. Instead, we utilized the BRICS built-in function "FindBRICSBonds" to identify decomposable bond, followed by "BreakBRICSBonds" to facilitate their decomposition.

*4、 At the beginning of the Section “**Network visualization of the DDiI predictions”, it is not clear what the authors mean by " We remove the known DDiIs used in the prediction model…". Do they perform again the same pipeline without considering the known drug-disease associations? Do they delete the known associations from the outcome they've already had in Section ‘Performance comparison’?*

Response: In section “Network visualization of the DDiI predictions” we aim to verify the model's ability to identify new interactions, so we remove the known interactions previously utilized for training to perform visual analysis.

*Minor comments:
1、In the results shown in Figure 7, there seems to be no* *“Disease/Side effect” node type, and the color marked in the figure seems to be the same as the “Target” node.*

Response: Thank you very much for your comments on the figure. The color schemes for the two node types, "Disease/Side Effect" and "Target," were too similar, so we have revised Figure 7 to ensure a clearer visualization." The **Figure 7** has been modified.

*2、 Some important works have not been* *reviewed or mentioned, such as doi:* *10.1093/bioinformatics/btac579,* *doi: 10.1093/bioinformatics/btab651,* *doi:* *10.1093/bioinformatics/btz718，**doi：**10.1371/journal.pcbi.1011382. Sufficient literature is important for potential readers.*

Response: Thank you very much for your suggestion. The work (doi: 10.1093/bioinformatics/btac579) has been introduced in “Related work” section.

We have added reviews for work (doi: 10.1093/bioinformatics/btab651) in “Related work” section, as “Fu et al. develop a novel link prediction model, multi-view graph convolutional network (MVGCN) for link prediction in biomedical bipartite networks.”

We have added reviews for work (10.1093/bioinformatics/btz718) in “Introductions” section, as “Yue et al. select 11 representative graph embedding methods and conduct a systematic comparison on 3 important biomedical link prediction tasks and 2 node classification tasks.”

We have added reviews for work (doi：10.1371/journal.pcbi.1011382) in “Introductions” section, as “Liu et al. present a subcomponent-guided deep learning method for accurate and interpretable CDR prediction, named SubCDR, to recognize the most relevant subcomponents driving response outcomes. However, this method only considers the substructure information of the drug without combining this substructure with biomedical network information.”
